# Preoperative Radiotherapy Does Not Increase the Risk for Early Complications Following Surgery for Oral Cancer: A Study on Data From the Randomized ARTSCAN 2 Trial

**DOI:** 10.1177/19160216251345473

**Published:** 2025-06-14

**Authors:** Kristin Carlwig, Maria Gebre-Medhin, Lennart Greiff, Peter Hällman, Per Nilsson, Johan Wennerberg, Björn Zackrisson, Johanna Sjövall

**Affiliations:** 1 Department of ORL, Head & Neck Surgery, Skåne University Hospital, Lund, Sweden; 2 Department of Clinical Sciences, Lund University, Lund, Sweden; 3 Department of Hematology, Oncology & Radiation Physics, Skåne University Hospital, Lund, Sweden; 4 Department of Clinical Sciences, ORL, Umeå University Hospital, Umeå, Sweden; 5 Department of Diagnostics and Intervention, Oncology, Umeå University, Umeå, Sweden

**Keywords:** oral cancer, squamous cell carcinoma of head and neck, radiotherapy, neoadjuvant therapy, postoperative complications

## Abstract

**Importance:**

The management of complications following oral cavity squamous cell carcinoma (OCSCC) surgery can be challenging. Previous studies show conflicting results on complication risks after preoperative radiotherapy (RT), necessitating a randomized controlled trial (RCT).

**Objective:**

To compare early complications during hospitalization for OCSCC surgery between patients receiving preoperative accelerated fractionated RT and those planned for but not yet exposed to RT.

**Design:**

A part of the ARTSCAN 2 RCT comparing preoperative accelerated RT with postoperative conventionally fractionated RT for OCSCC.

**Setting:**

A multicentre trial in 6 tertiary care hospitals in Sweden.

**Participants:**

Untreated and resectable OCSCC patients of all stages recommended combination treatment by the local multidisciplinary board.

**Intervention:**

Preoperative accelerated RT was administered twice daily to a total dose of 68 Gy, completed 4 to 6 weeks before surgery.

**Main Outcome Measures:**

Complications during hospitalization included wound infection, neck flap necrosis, chyle leakage, oro/pharyngocutaneous fistula, free flap necrosis, tracheostomy, revision surgery, and medical complications. Length of surgery, perioperative blood loss, and transfusions were also monitored.

**Results:**

Two hundred and twenty-one patients were eligible for analysis: 103 in the preoperative RT group and 118 not yet exposed to RT. Complication rates were low, with no statistically significant differences between groups. Patients receiving preoperative RT had similar wound infection rates (12/103; 11.7%) to those not exposed (9/118; 7.6%) (*P* = .31). Among free flap patients, 1/40 (2.5%) in the preoperative RT group and 3/52 (5.8%) in the unirradiated group had free flap necrosis (*P* = .63). No differences were found in oro/pharyngocutaneous fistula frequency (3/103; 2.9% vs 3/118; 2.5%) (*P* = 1.00).

**Conclusion and Relevance:**

Preoperative accelerated RT at 68 Gy, administered 4 to 6 weeks before surgery, does not increase early complications. Although survival rates, morbidities, quality of life, and societal costs need consideration in the ARTSCAN 2 assessment, our findings show that early postoperative complication risks remain unchanged by preoperative RT.

**Trial Registration:**

ISRCTN, ISRCTN00608410, Registered 20 March 2008—Retrospectively registered, https://www.isrctn.com/ISRCTN00608410.

## Graphical abstract

**Graphical Abstract:**
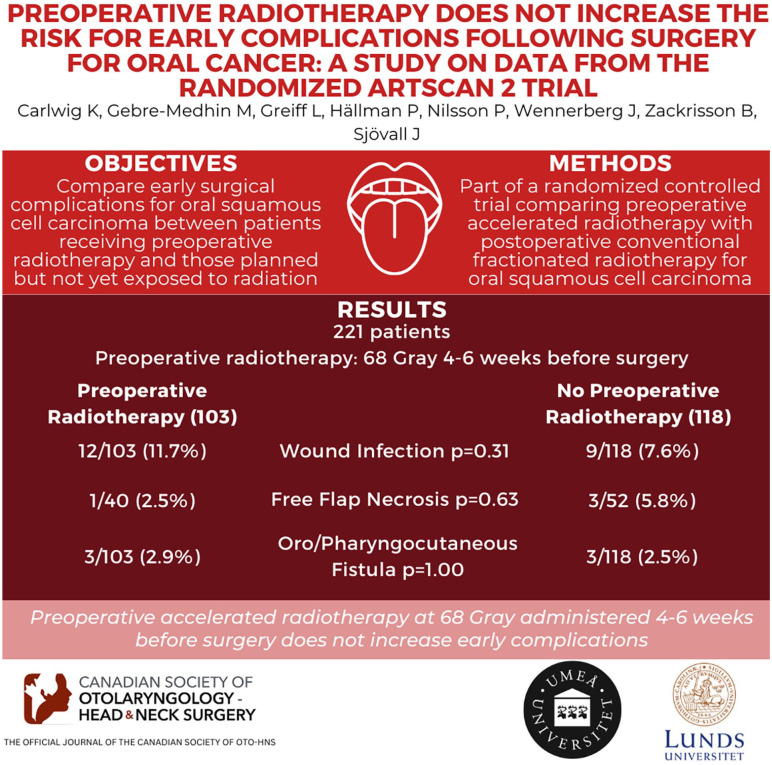

## Introduction

Patients with resectable oral cavity squamous cell carcinoma (OCSCC) are frequently offered a combination of surgery and radiotherapy (RT). RT produces both acute and long-term effects. When the acute inflammatory response, reflected as edema, dermatitis, and mucositis, subsides, tissue fibrosis with decreased vascular perfusion develops.^1-3^ Inflammation as well as fibrosis may affect the surgical outcome, resulting in complications. Accordingly, it is debated whether or not RT is best given before or after surgery, for example, when a combination of these measures is considered for OCSCC.^4-6^ However, in this context, randomized controlled trials (RCT) focusing on OCSCC are scarce.^7^

Previous studies on types and rates of complications following surgery for head and neck cancer performed after RT are conflicting. Some report more complications,^8-14^ including flap loss,^11,15-17^ while others show no differences (cf. non-RT patients).^9,10,13,16,18-21^ However, a majority of the studies are retrospective^8-10,16-21^ and criteria for occurrence and type of complication differ. Furthermore, the time interval from RT to surgery varies as well as the RT dose given.^10,17-20^ Moreover, the surgery is sometimes conducted in salvage situations rather than as part of a planned combination treatment.^8,18,21^ Finally, these studies do not focus specifically on OCSCC.^8,9,16,18-21^

The ARTSCAN 2 study is a RCT involving patients with OCSCC subjected to combinations of surgery and RT. The patients were randomized to preoperative accelerated RT or postoperative conventionally fractionated RT. At 5 years follow-up, no statistically significant difference in overall survival was observed between the treatment groups. In the primary publication, locoregional control, toxicity during RT and long-term morbidity were also described.^7^ Here, we report on perioperative complications in the treatment groups of the ARTSCAN 2 trial, that is, in recently irradiated patients versus unirradiated patients, with the hypothesis that complications may be more common following preoperative RT. The analysis also includes comparisons of the length of surgery, perioperative blood loss, blood transfusions, and the need for a tracheostomy tube.

## Methods

### Study Design

ARTSCAN 2 is a multicenter, open-label RCT^7^ that was conducted in Sweden from 2008 to 2016. Patients intended for a combination treatment of OCSCC were randomized 1:1 to preoperative accelerated fractionated RT or postoperative conventionally fractionated RT. Randomization was stratified by study center, tumor subsite, and clinical stage. In the present analysis, focusing on early complications (ie, during hospitalization following surgery), both treatment groups of ARTSCAN 2 were used, allowing for comparisons between patients who had received preoperative accelerated RT and those who had not yet received RT (in this study indicated in tables as “no RT”). In addition, we have extracted data from the cohort of the Southern Healthcare Region of Sweden, accounting for a third of the included patients, for two of the analyses [preoperative Hemoglobin (Hb) levels and length of hospitalization]. The study was approved by the Ethics Board (Umeå: 07-178M), and written informed consent was obtained.

### Patients

Patients over the age of 18 years with resectable and previously untreated OCSCC of all stages, but without distant metastases were considered for ARTSCAN 2. Each center offered participation to patients who were recommended combination treatment by the local multidisciplinary board. Patients with previous head and neck cancer were excluded. Tumors were staged according to the seventh edition of the UICC’s TNM classification. Detailed information on the methodology of ARTSCAN 2 has previously been presented.^7^

### Surgery

In the preoperative RT group, tumor borders were marked with coal suspension to aid adequate later surgery aiming at a 10 mm macroscopical margin. Accordingly, independent of the tumor response to preoperative RT, the marked margin guided the surgical resection. Surgery was performed within 4 to 6 weeks after RT. Neck dissections were performed on node-positive patients, while node-negative patients were not mandatorily recommended the procedure. Early (ie, specifically and only during the hospitalization period following surgery) complications were recorded: bleeding during the surgical procedure, wound infection, skin necrosis of the neck flap, chyle leakage, oro/pharyngocutaneous fistula, free flap necrosis, need for revision surgery, and medical complications (ie, myocardial ischemia, deep vein thrombosis, pulmonary embolism, airway infection). In addition, the length of surgery, perioperative blood loss, blood transfusions, and the need for a tracheostomy tube were monitored. Finally, preoperative Hb levels and the length of hospitalization were analyzed for patients of the Southern Healthcare Region of Sweden receiving surgery at Skåne University Hospital.

### Radiotherapy

Preoperative accelerated RT was given twice daily (1.1+2 Gy per fraction) with a concomitant boost technique for 5 days per week for 4.5 weeks to a total dose of 68 Gy to the primary tumor and lymph node metastases. The interfraction interval was not less than 7 hours. Elective lymph nodes were treated with 46 Gy in 23 fractions. For all midline and/or node-positive tumors, the neck was bilaterally treated. In lateral T1-2 and N0 tumors, unilateral lymph node treatment was allowed.

### Statistical Analysis

Continuous variables with normal distribution were presented as mean with standard deviation (SD). Continuous variables with non-normal distribution were reported as median with minimum (min.) or maximum (max.) or first to third quartile range (Q1-Q3). Categorical variables were presented as frequencies and percentages. For ordinal data, the Mann–Whitney *U* test was used. Differences between groups were evaluated by the *t*-test (normal distribution) or the Mann–Whitney *U* test (non-normal distribution). Pearson’s chi-square test or Fisher’s exact test were used for categorical variables. *P*-values <.05 were considered statistically significant. Analyses were performed using version 26.0 of the SPSS statistics program (IBM, Armonk, NY).

## Results

**Figure 1.**
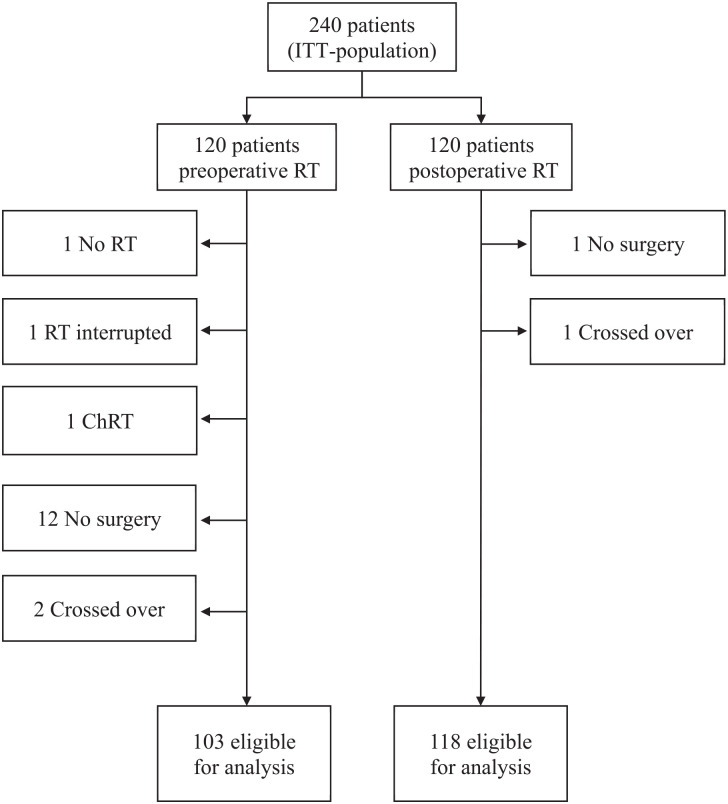
Flow chart illustrating patients eligible for analysis of surgical and medical complications. ITT, intention to treat; RT, radiotherapy; ChRT, chemoradiotherapy.

Out of the 240 patients eligible for intention-to-treat analysis in ARTSCAN 2, 19 patients were excluded, leaving 221 patients eligible for the present analysis: 103 in the preoperative RT group and 118 in the group that had not yet received RT (Figure 1). Twelve patients in the preoperative RT group were excluded because they never had surgery: 3 patients died during/after RT, 2 patients were diagnosed with a synchronous cancer, 2 developed comorbidities and were considered in remission, 2 suffered from severe toxicity from RT and were considered in remission, 1 patient had severe toxicity and was excluded from surgery on own request, and 2 patients developed fast tumor growth progression and were assessed as nonresectable. Baseline characteristics were well balanced between the groups. The median age was 66 years and 62% were males. Sixty-nine per cent of the tumors were tongue or floor-of-mouth cancers (Table 1).

**Table 1.** Baseline Demographics and Clinical Characteristics.

Patient characteristics	Preop. RT (n = 103)	No RT (n = 118)
Age, median (min.-max.)	65 (31-84)	67 (23-85)
Gender, n (%)		
Male	67 (65)	70 (59)
Female	36 (35)	48 (41)
WHO performance status, n (%)		
0	91 (88)	104 (88)
1	8 (8)	12 (10)
2	4 (4)	1 (1)
3	0 (0)	1 (1)
Smoker,^a^ n (%)		
Never	34 (34)	50 (43)
Active	31 (31)	19 (16)
Previous	36 (36)	48 (41)
Hb at randomization, mean (SD)	141 (12)^b^	140 (12)
Primary tumor site, n (%)		
Tongue/Floor of mouth	70 (68)	82 (69)
Gingiva + other sites	33 (32)	36 (31)
T stage, n (%)		
T1	13 (13)	13 (11)
T2	58 (56)	62 (53)
T3	9 (9)	17 (14)
T4	23 (22)	26 (22)
Nodal status, n (%)		
N0	72 (70)	88 (75)
N1	12 (12)	11 (9)
N2a	0 (0)	0 (0)
N2b	15 (15)	16 (14)
N2c	4 (4)	3 (3)
N3	0 (0)	0 (0)
Clinical stage, n (%)		
I-II	51 (50)	60 (51)
III-IV	52 (50)	58 (49)

Abbreviation: RT, radiotherapy.

a Two missing values in Preop RT, 1 in No RT.

b Two missing values.

Postoperative complication rates were low in general. Wound infection occurred in 10% of the patients and was the most common complication, with no differences between the study groups. Sixty-five and 79 patients were neck dissected in the preoperative RT group and the group that had not yet received RT, respectively (Table 2). Among neck-dissected patients, 2 developed skin necrosis of the neck flap, both in the preoperative RT group (*P* = .20). No patient had chyle leakage. Medical complications were uncommon, with no differences between the groups (Table 3). Thirty-day postoperative mortality concerned a single patient who belonged to the group not yet exposed to RT, who died 19 days after surgery related to a cardiopulmonary arrest.

**Table 2.** Types of Surgery Performed.

Surgical procedure	Preop. RT (n = 103)	No RT (n = 118)
Tumor resection, n (%)	All	All
Free flap, n (%)	40 (39)	52 (44)
Local flap, n (%)	4 (4)	3 (3)
Neck dissection, n (%)	65 (63)	79 (67)

Abbreviation: RT, radiotherapy.

**Table 3.** Early Postoperative Complications.

Complications	Preop. RT (n = 103)	No RT (n = 118)	*P*-value
Acute reoperation needed, n (%)	8 (8)	12 (10)	.53
Post op. blood transfusion, n (%)	9 (9)	15 (13)	.34
Wound infection, n (%)	12 (12)	9 (8)	.31
Oro/pharyngocutaneous fistula, n (%)	3 (3)	3 (3)	1.00
Myocardial ischemia, n (%)	1 (1)	0 (0)	.47
Deep vein thrombosis, n (%)	0 (0)	0 (0)	—
Pulmonary embolism, n (%)	0 (0)	1 (1)	1.00
Airway infection, n (%)	1 (1)	2 (2)	1.00

Abbreviation: RT, radiotherapy.

Ninety-two microvascular free flap reconstructions were performed, 40 in the preoperative RT group and 52 in the group that had not yet received RT. Seven local flaps were conducted, 4 in the preoperative and 3 in the group that had not yet received RT (Table 2). Among the 99 flap-operated patients, there were 6 flap necroses: 2 in the preoperative and 4 in the group that had not yet received RT (*P* = .69). Four of the flap necroses occurred in free flaps (4/92; 4.3%), 1 in the preoperative RT group (1/40; 2.5%) and 3 in the group that had not yet received RT (3/52; 5.8%) (*P* = .63).

The mean time for the surgical procedure was 350 minutes for the study population as a whole, with no statistically significant difference between the study groups (Table 4). Median operation time excluding the free flap-operated patients from the analysis (60 vs 64 patients, missing data n = 5) was 150 versus 131 minutes (*P* = .70), respectively, for the preoperative group and for the group that had not yet received RT. The corresponding numbers for the free flap-operated patients (40 vs 50 patients, missing data n = 2) was 570 versus 600 minutes (*P* = .63). Among the neck-dissected patients (64 vs 76 patients, missing data n = 4), the corresponding numbers were 492 versus 523 minutes (*P* = .95).

**Table 4.** Perioperative Parameters.

Parameters	Preop. RT (n = 103)	No RT (n = 118)	*P*-value
Surgery duration, min, mean (SD)	344 (250)^a^	357 (254)^b^	.70
Blood loss, ml, median (Q1-Q3)	300 (100-563)^c^	250 (138-500)^d^	.78
Intra op. blood transfusion, n (%)	24 (24)^e^	14 (12)	.022
Blood units, median (min.-max.)	2 (1-6)	2 (1-7)	.46
Tracheostomy tube, n (%)			.99
No tube	60 (58)	67 (57)	
<14 days	36 (35)	47(40)	
≥14 days	7 (7)	4 (3)	

Abbreviations: Q1, first quartile; Q3, third quartile; RT, radiotherapy; SD, standard deviation.

a Three missing values.

b Four missing values.

c Eight missing values.

d Three missing values.

e One missing value.

There was no significant difference in perioperative bleeding, but significantly more patients received a blood transfusion in the preoperative RT group (24/102 vs 14/118, *P* = .022) (Table 4). The mean preoperative Hb level, analyzed for the southern patient cohort, was 124 and 141 g/L, in the preoperative group and in the group that had not yet received RT, respectively (*P* < .001). The median hospitalization time for the southern patient cohort (n = 75), was 19 days (Q1-Q3: 9-24) for the preoperative RT group and 15 days (Q1-Q3: 8-20) for the unirradiated group (*P* = .38).

## Discussion

In this study, involving patients offered a combination of surgery and RT for OCSCC in an RCT, we assessed early complications following surgery and compared patients exposed to preoperative RT with those not yet exposed to any RT. Our hypothesis that early surgical and medical complications might be more common following preoperative RT was not confirmed. Indeed, the complication rates, which in general were low, did not differ between the study groups in this RCT.

Previous studies on types and rates of complications following surgery performed after RT are conflicting.^8-10,12-21^ However, in a recent meta-analysis on free flap outcome in head and neck cancer, previous RT was identified as a statistically significant risk factor for free flap failure and other complications.^11^ Accordingly, it was concluded that: “These high-morbidity complications must be taken into consideration when determining which patients should receive neoadjuvant radiation therapy.” Obviously, the meta-analyzed studies represented a mix of patients exposed to RT to the head and neck region, for example, not only as part of a planned combination treatment. Furthermore, the RT dose and the duration between RT and surgery were not uniform. In contrast, in this study, we focused specifically on combination treatment of OCSCC and observed no differences in early surgical and medical complications between the preoperative RT group and the group that had not yet received RT. It must be underscored that the conclusion is valid specifically for OCSCC and when preoperative RT is given to a dose of 68 Gy and finalized 4 to 6 weeks prior to the surgical procedure.

In this analysis of the ARTSCAN 2 data, we focused exclusively on early (ie, during hospitalization following surgery) surgical and medical complications. In the primary report of the ARTSCAN 2 study, we observed late morbidities with significantly more events of weight loss, laryngeal mucosa alterations, and osteoradionecrosis in the preoperative accelerated RT group cf. the postoperative conventionally fractionated RT group.^7^ Furthermore, in a previous retrospective study, we have demonstrated a close to 10-fold greater complication rate at the mandibulotomy site, that is, oro-cutaneous fistulas, bone exposure, and nonunion, for preoperative RT (cf. postoperative RT) in patients where the measure was undertaken as part of the surgical procedure to gain access to the tumor site.^22^ Clearly, it is important to consider both the risk of early and late complications when deciding to position the RT ahead of the surgery for OCSCC, along with obvious aspects such as survival and quality of life, as well as societal costs. Previous analyses of the ARTSCAN 2 data have reported on survival, locoregional control, toxicity during RT, late toxicity,^7^ and societal costs.^23^ An analysis of quality of life and cost-utility is ongoing.

Previous studies suggest that the optimal time for flap survival is when surgery is performed within 6 weeks after RT.^15,16^ Furthermore, with an increased time elapsed between RT and surgery (>6 weeks), significantly more complications emerge (local infections, delayed wound healing).^16^ In a retrospective study by Goguen et al on complications related to the timing of neck dissections, that is, <12 weeks versus >12 weeks (range 4-26 weeks) after completion of chemoradiotherapy (ChRT), there were significantly more patients with at least one event in the >12 weeks group.^24^ Arguably, such observations may relate to the tissue response to RT: Schultze-Mosgau et al reported a reduction in vascularization, increase in fibrosis, and decrease in the capillary lumen with prior irradiation (60-70 Gy, 1-7 years before surgery) cf. neoadjuvant ChRT (40-50 Gy and cisplatin/5-FU) followed by microvascular/free flap surgery 6 weeks later, which was paralleled by a higher free flap failure rate in the former group.^25^ Accordingly, the interval between RT and surgery, and possibly the RT dose delivered, are potential factors of flap survival. In agreement, it has been demonstrated that radiation-induced fibrosis and reduced vascularity occur 4 to 12 months after RT and progress over time.^26^ Conversely, our low numbers of free flap failures and postoperative complications may be explained by the short interval between preoperative RT and surgery.

Of the early surgical complications assessed in this study, free flap failure may be the most severe. It increases the surgical trauma, prolongs hospitalization, increases morbidity, and often requires additional advanced actions. Accordingly, a majority of previous studies on surgical complications associated with preoperative RT concern free flaps. The literature provides mixed evidence, with some studies reporting a significant relationship between previous RT and free flap failure.^16,17,27^ In our study, with data from an RCT, the overall free flap failure rate was 4.3%, which corresponds well with previous reports on failure rates ranging from 2% to 10%.^8,9,17-20,27,28^ No statistically significant difference in free flap failure between the preoperative RT group and the group that had not yet received RT was observed. This finding is in agreement with previous retrospective studies on free flap failures in patients exposed to preoperative RT (cf. no preoperative RT).^9,19,20^

Our results are in accordance with a previous RCT on head neck cancer, including OCSCC, studying the occurrence of fistulas, delayed healing, carotid blow-outs, and free flap failures in patients exposed to RT (50 Gy in 5 weeks, 2 Gy per fraction) followed by surgery 4 to 6 weeks later (cf. no preoperative RT). The time duration when complications were monitored was not stated. In the subgroup analysis of oral cancers (n = 30), the result remained insignificant.^29^

Perioperative blood loss and transfusions were monitored in this study as potential markers of complications. There was no difference in blood loss between the preoperative RT group and the group not yet exposed to RT, but significantly more patients in the preoperative RT group received blood transfusions. The finding led to the question whether the preoperative Hb levels differed between the study groups. The Hb levels were found to be lower in the preoperative RT group, reflecting a possible anemia-producing effect of RT.^30^ Given the main results of this study, that is, a lack of effects of preoperative RT on early surgical and medical complications, the present observation on preoperative Hb may add little. However, our observation in OCSCC that perioperative transfusions did not associate with complications following preoperative RT, is in contrast to, for example, observations in esophageal cancer where this seems to be the case.^31^

### Limitations

The ARTSCAN 2 study recorded only complications during the hospitalization period. A 30-day morbidity period, covering outpatient data and re-admissions could have revealed more complications. Moreover, data on preoperative Hb levels and the length of stay at the hospital were restricted to a subgroup of approximately 30% of the patients.

### Conclusion

Preoperative accelerated RT for OCSCC, given to a dose of 68 Gy and finalized 4 to 6 weeks prior to a surgical procedure, does not affect early surgical or medical complication rates. While data on survival, locoregional control, long-term morbidity, quality of life, and societal costs must be integrated into the global assessment of the clinical applicability of the ARTSCAN 2 RCT, the present results suggest that the risks for early postoperative complications are not affected by the treatment order when surgery is combined with radiotherapy for OCSCC.
